# From Structure to Function: Mapping the Connection Matrix of the Human Brain

**DOI:** 10.1371/journal.pbio.0060164

**Published:** 2008-07-01

**Authors:** Liza Gross

When American architect Louis Sullivan proffered the enduring mantra of 20th century design—form follows function—he chided his peers for violating in art a law so clearly visible in the “open apple blossom” and “sweeping eagle in his flight.” The notion that the essence of things takes shape in the matter of things, first articulated in Aristotle's philosophy, has long guided biologists' attempts to understand the inner workings of the most complex organ known—the human brain. Fine-grained descriptions of anatomical features of the eye and ear, for example, have yielded critical insights into the neural basis of image and sound perception. But for a systems-level understanding of how the brain works, researchers look to the overall topology of network connections among neurons for answers.

The brain gathers, extracts, and stores salient details about our environment to plan and carry out behaviors that are appropriate (if all goes well) to a given situation. Increasingly, researchers view these perceptual and behavioral responses as computational problems solved by networks made up of more than a hundred billion neurons connected through some hundred trillion synapses. Computational neuroscientists rely on mathematical tools and computer modeling to study how functional groups of neurons interact and how they process, store, and transmit information to mediate human cognition and behavior. Any attempt to understand how a network functions, however, depends on identifying its elements and how they connect to form functional modules. As advances in technology allow researchers to produce clearer pictures of the architecture of neural circuits, new approaches in network analysis and graph theory offer novel insights into how brain networks operate.

In a new study, Patric Hagmann, Olaf Sporns, and their colleagues mapped the large-scale network structure of the nerve fibers linking each region of the human cerebral cortex—the highly furrowed outer surface of the brain responsible for higher cognitive functions. Using structural and functional brain imaging technology, combined with computational analyses, the researchers identified highly connected, centrally located regions of the human cortex. These hubs form a “structural core” of the human brain, which the researchers think may act as central processors, integrating multiple inputs across the cortex. Intriguingly, these hubs correspond to a recently reported “default network,” a neural system that shows increased activity levels when subjects are resting.

**Figure:**
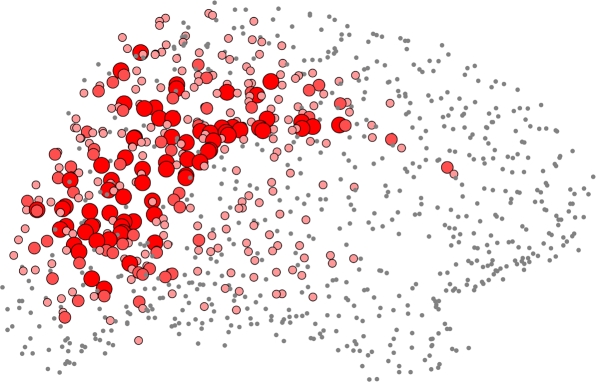
Structural and functional brain imaging analyses, combined with computational analyses, reveal highly connected, centrally located regions of the human cortex that form a “structural core” of the brain.

In brains of five healthy human participants, the researchers mapped the network of anatomical connections among the cortex's white matter tracts, the axon fibers that relay messages between neurons, using state-of-the-art diffusion magnetic resonance imaging (MRI) technology. Diffusion MRI is a noninvasive scanning technique that estimates fiber connection trajectories based on gradient maps of the diffusion of water molecules through brain tissue. Recent innovations have yielded a highly sensitive variant of the method, called diffusion spectrum imaging (DSI), which can accurately depict the orientation of multiple fibers that cross a single location. In this paper, the technique is applied for the first time to the whole human cortex.

With the data generated by DSI, the researchers generated a structural connection matrix of the entire brain, divided into 66 cortical anatomical regions and some 1,000 regions of interest (ROIs). They characterized the properties of the global and regional connection matrices using network analysis, based on estimates of elevated node “degree” (fiber counts) and node strength (fiber density), to locate structural hubs across the brain and within local regions. These computational analyses identified several ROIs with a high number of dense connections within subregions of the cortex, ultimately revealing a network core—a set of highly, mutually interconnected nodes—composed of subregions within the parietal and posterior medial cortical regions, including components associated with the “default network” that becomes activated when a person is resting. The fact that these regions span both hemispheres of the brain, the researchers argue, suggests that the structural core may serve as a central integrating system, coordinating processes across segregated regions in both hemispheres.

To investigate the potential role this structural core may serve, the researchers used functional MRI (fMRI) to record brain activity from their five participants, who were asked to rest quietly while remaining alert. The researchers found a significant correlation between the patterns of structural connections estimated by DSI and the functional interactions measured by fMRI among all cortical regions. This correlation indicates that the structure of brain connections shapes the ways in which brain regions interact and exchange signals.

This study represents a major step toward mapping the connectivity patterns of the billions and billions of neurons whose interactions allow us to navigate the daily demands of human existence. The next major challenge will be to incorporate the brain's subcortical regions, such as the thalamus and basal ganglia, into a complete human “connectome” for structurally guided investigations of brain function. Until then, future studies can further explore the relationships between structure and function using the approach described here. And by comparing differences in structural and functional connectivity between individuals, researchers can begin to identify the neural basis of variation in human behavior.

